# Philadelphia Hospital, Blockley

**Published:** 1859-09

**Authors:** 


PHILADELPHIA HOSPITAL, BLOCKLEY.—The office of Chief Resident Physician to this institution having been abolished, the Board of Guardians, at their meeting on the eighth of August, elected the following gentlemen as Visiting Physicians, Surgeons, and Obstetricians:—

*Physicians.*—Drs. J. L. Ludlow, W. Mayburry, C. P. Tutt, and F. E. Luckett.

*Surgeons.*—Drs. R. J. Levis, D. H. Agnew, S. D. Gross, and R. S. Kenderdine.

*Obstetricians.*—Drs. R. A. F. Penrose, L. D. Harlow, W. D. Stroud, and J. Wiltbank.

